# Assessing the Effectiveness of a Massive Open Online Course for Caregivers Amid the COVID-19 Pandemic: Methodological Study

**DOI:** 10.2196/48398

**Published:** 2023-09-25

**Authors:** Maria José Lumini, Maria Rui Sousa, Berta Salazar, Teresa Martins

**Affiliations:** 1 Nursing School of Porto Centro de Investigação em Tecnologias e Serviços de Saúde (CINTESIS) Rede de Investigação em Saúde (RISE) Porto Portugal

**Keywords:** caregivers, education, COVID-19, distance, effectiveness, skill, safe care, health system, older people, family, social isolation, massive open online courses, care challenges

## Abstract

**Background:**

The COVID-19 pandemic has presented significant challenges to health care systems, particularly impacting the older population due to their vulnerability and increased susceptibility to severe complications. Many of the most vulnerable individuals rely on informal caregivers, who play a vital role in enabling them to continue living in their homes. However, social isolation and limited access to health services during the pandemic have made caregiving more difficult. In response, massive open online courses (MOOCs) have emerged as a training and support solution for caregivers. This study focuses on a MOOC developed to assist caregivers during the pandemic, aiming to enhance their knowledge of COVID-19 and prevention measures and promote effective self-care practices.

**Objective:**

The study’s aim is to develop and validate a MOOC integrating personal and housing hygiene measures to be adopted in self-care–related activities, surveillance, and monitoring by caregivers of the most vulnerable home-dwelling–dependent people, to provide safe care and prevent SARS-CoV-2 infection.

**Methods:**

A methodological study was developed. The content of the MOOC was developed based on scientific evidence and a Delphi study. The course was organized into 9 modules, addressing aspects related to safe self-care assistance and minimizing the risk of contagion. A convenience sample of 33 informal caregivers was recruited through a caregivers’ association to verify the adequacy of the course. Knowledge questionnaires were administered before and after the course to assess the impact on caregivers‘ knowledge. The Family Caregiving Factors Inventory was used to evaluate caregiver resources, knowledge, expectations, and difficulties. Additionally, the technology acceptance model was applied to assess participants’ satisfaction with the MOOC.

**Results:**

Prior to attending the MOOC, participants demonstrated an average knowledge level score of mean 14.94 (SD 2.72). After completing the course, this score significantly increased to mean 16.52 (SD 2.28), indicating an improvement in knowledge. Caregivers found the course accessible, valuable, and applicable to their caregiving roles. Feedback regarding the MOOC’s structure, illustrative videos, and language was overwhelmingly positive. Participants perceived the course as a valuable resource for decision-making in care delivery, leading to enhanced self-esteem and confidence.

**Conclusions:**

The MOOC has proven to be an effective tool for increasing caregivers’ knowledge and empowering them in their roles. Remarkably, even low-literacy caregivers found the course valuable for its clear and understandable information. The MOOC demonstrated its adaptability to challenges faced during the pandemic, ensuring access to relevant information. This empowering strategy for caregivers has yielded positive outcomes. The MOOC represents a tool to support and empower informal caregivers, enabling them to provide optimal care during difficult times.

## Introduction

The COVID-19 disease caused by the SARS-CoV-2 virus posed an enormous challenge to health systems and led to increased morbidity and mortality in the general population. The World Health Organization (WHO) [1] reports that as of January 2022, close to 271.2 million new cases and 2.2 million deaths in the European region were caused by the disease. Moreover, from October to December 2022, the weekly average of deaths and cases decreased by 31% and 17%, respectively [1]. In the WHO European Region, in the first week of February, 323,873 new confirmed cases of COVID-19 and 1670 new deaths were reported by the national authorities [2]. Data from the first week of February 2023 suggest that 27.9% of cases were related to persons older than 65 years, and 90.8% of fatal cases concerned persons older than 65 years [2].

Although all age groups are at risk of contracting COVID-19, older people are more prone to develop serious conditions associated with the disease [2]. In addition, this age group is more vulnerable because of their frailty resulting from aging [3]. For example, their immune system is more likely to weaken if they contract the disease, leading to severe associated complications with less favorable outcomes [4].

However, physical and mental vulnerability is not solely a product of aging. Also, people with multimorbidities who live in unfavorable social and economic contexts, have restricted access to health care, and have disabilities or are in a dependent situation are exposed to greater vulnerability [4].

To counter this trend, the WHO [1] recommends implementing personal and daily measures to curb the spread of the virus, coupled with other directives. It also emphasizes careful approaches to older people, their families, and their caregivers during the pandemic. Due to their extremely frail condition, older people need to maintain a healthy diet, take medication to ensure health and well-being, and have access to social resources. Thus, the focus should be on providing accurate and clear information so that patients and their caregivers understand how to stay physically and mentally healthy during the pandemic and what to do if they fall ill [3].

In Portugal, data released in a report [5] pointed to 110,355 home-dwelling adults who are partially unable to care for themselves, of which 48,454 were totally dependent. Also, estimates point to 1.4 million informal caregivers in Portugal [6]. It is widely known that informal caregivers lack information about caring for the most vulnerable people in a home context. Most of these people depend on care from family members who are often poorly prepared and lack support from health professionals. However, there is a gap in what to do to empower these caregivers to face the new challenges posed by the pandemic.

Various pedagogical alternatives are available to prepare nurses to deliver better patient and family support. In this regard, introducing digital solutions is crucial since they contribute to increasing access to information and represent a substantial part of the nursing care transformation plan. Nowadays, information and communication technologies intersect all educational environments and are essential to nurses’ daily interventions. Recent technological advancements have greatly influenced educational strategies in nursing. The pandemic prompted the revision of teaching and learning methods. Therefore, traditional education has been gradually superseded or supplemented by distance education, supported by virtual classes and other web-based tools [7,8].

Massive open online courses (MOOCs) have emerged as a solution to support the empowerment of family caregivers of vulnerable people, especially in times of social isolation. These courses are presented as a new pedagogical approach that helps overcome learning barriers. MOOCs incorporate a social model, a distributed network approach with significant user autonomy, and are aimed at adults interested in lifelong learning opportunities within the scope of continuous training [9]. The paradigm shift and the growth of MOOCs are a reality derived from the increase in web-based learning and e-learning. As the conceptualization of MOOCs begins to transform, variations will be adapted to unique contexts, focusing on the needs of the target audience and human connections [9]. MOOCs empower caregivers through comprehensive expert-led courses, interactive learning, global communities, and flexible schedules. Unlike other asynchronous methods, MOOCs offer tailored content, verified certificates, continuous support, and practical simulations, enhancing caregivers’ skills and knowledge for better caregiving outcomes and personal growth [10,11].

Thus, this study sought to contribute to the empowerment of the caregiver by developing and validating a MOOC and integrating personal and housing hygiene measures into self-care–related activities, as well as surveillance and monitoring by caregivers of vulnerable home-dwelling–dependent people to provide safe care and prevent SARS-CoV-2 infection.

## Methods

### Development of the MOOC

A set of topics considered relevant for the caregiver were selected, such as COVID-19 prevention measures and content related to the provision of care to dependent people as self-care, namely, feeding and hydration, positioning and transfer, hygiene care, prevention of pressure injuries, prevention of falls, and medication management. The content production started by designing a plan considering the pandemic situation and was grounded on scientific evidence. The MOOC was divided into nine modules: (1) presentation of the course, (2) COVID-19 prevention measures, (3) feeding and hydration, (4) positioning and transfer, (5) hygiene care, (6) dressing or undressing, (7) prevention of pressure injuries, (8) fall prevention, and (9) medication.

The contents related to COVID-19 prevention measures were developed specifically for this MOOC. Other content previously developed and integrated into a digital platform [12] was also used.

This new content design followed the recommendations of the plain language method, comprising 4 steps: adaptation to the target population, language, and style; organization; layout and design [13]; and the Delphi study. A panel of experts was invited to conduct the Delphi study. This study involves a group of experts allowing the formulation of judgments to evaluate and classify a set of ideas on specific issues [14]. The experts’ selection followed the recommendations of Boateng et al [15]. The experts were recruited from a task force appointed by a central hospital in the northern region of the country and a crisis cabinet created in the northern regional health administration to tackle the pandemic outbreak. The group of experts included a public physician, 6 public health nurse specialists, 2 specialists in infection prevention and control, a microbiologist, and an infectious disease specialist. A total of 17 questions were formulated (Textbox 1) with respective answer options, based on two documents: the “Strategic Preparedness and Response Plan” (created by the WHO) and the ”National Plan–Preparation and Response to Disease–New coronavirus (COVID-19)” (created by the Portuguese General-Directorate of Health). The questions were grounded on available scientific evidence. The questions were presented as a grid, with detailed guidelines for the measures to be integrated into the course written in simple language without technical terms to facilitate its understanding. The document was sent to the experts, who were asked to describe the relevance and clarity of each information unit by scoring them according to their importance and applicability (from 5, very important and clear, to 1, not or only slightly important and confusing). The experts were informed that the parameters with a score lower than 4 would be eliminated or reformulated according to the suggestions and comments received. A period of 3 weeks was set for the submission of the complete document.

The experts’ panel carefully addressed appropriate terminology, and modifications of syntax and lexicon were considered to facilitate understanding.

Each module was recorded on video in a studio, and the content was later edited with animations to further communicate the message. To facilitate learning, other pedagogical resources were also developed for each module, namely, a written synthesis of the contents was made available on a static web page, a questionnaire was provided so that participants could validate their knowledge after viewing the videos, and a support manual was distributed as a PDF.

**Figure 1:**
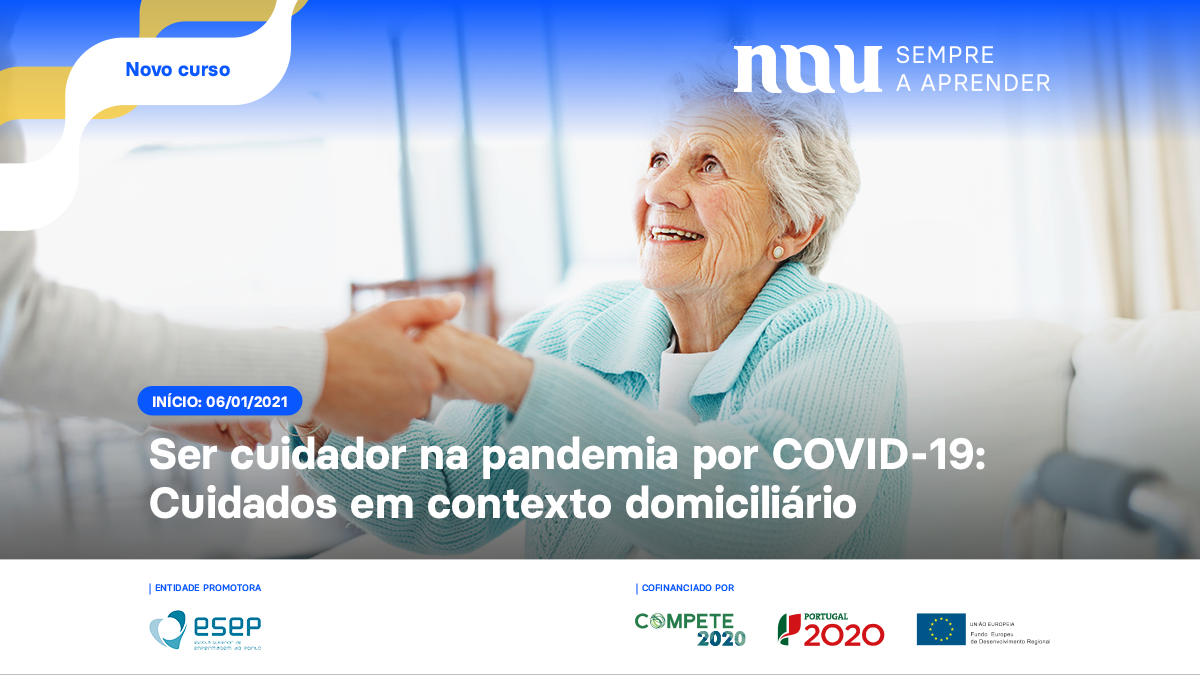
Massive open online course delivered in an open-access platform managed by the Scientific Computing Unit of the Portuguese Foundation for Science and Technology.

The MOOC was delivered on an open-access platform managed by the Scientific Computing Unit of the Portuguese Foundation for Science and Technology (Figure 1) and was available throughout 2021. This platform promotes digital development, inclusion and digital literacy, education, and qualification of the working population. It also allows the creation of courses in MOOC format, open and accessible to all, integrating the intersecting actions of the Portugal INCoDe.2030 initiative.

**Guiding questions sent to experts.**

**Questions**

- What is COVID-19?
- What are the symptoms of COVID-19?
- Is it only people with symptoms who transmit the disease?
- What is the incubation period of the disease (being the time it takes from one being infected to developing symptoms)?
- Is there a treatment for COVID-19?
- How is COVID-19 transmitted?
- What is community transmission?
- What are the prevention measures?
- Why and when should one wash their hands?
- What are the measures of social distancing?
- What are the measures of respiratory etiquette?
- What measures to take when being on public transportation?
- What personal protection measures to take when providing care?
- What measures to take when leaving home?
- What measures to take with housing hygiene?
- What surveillance and monitoring measures should one have with the family member?
- How to express affection and care without compromising the safety of the person receiving care?

### Study Design

**Figure 2:**
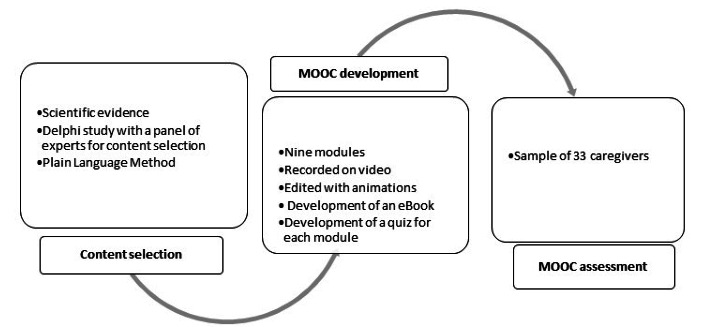
Study design. MOOC: massive open online courses.

A methodological study was developed. First, a Delphi study was carried out to validate the contents to be integrated into the MOOC. Subsequently, a pre- and posttest study was conducted with a convenience sample to verify the adequacy of the course to its target audience. The study design is shown in Figure 2.

### Participants

The participants who helped with the face validation of the MOOC were recruited through convenience sampling from the leading caregivers’ association of the region where the study was conducted. The inclusion criteria were (1) being an informal caregiver who agreed to participate in the study, (2) being aged 18 years or older, and (3) having access to home internet and skills in information technologies or otherwise having the support of a family member or significant other.

The caregivers’ association identified 52 informal caregivers who fulfilled the inclusion criteria to participate in the study. However, between recruitment and the beginning of the study, 19 caregivers withdrew because they were no longer available, had difficulties accessing the platform, or because the recipient of care died.

The characteristics of the 33 caregivers who participated in the assessment of the MOOC are shown in Table 1. The participants were aged between 37 and 78 years, with an average age of 53.5 (SD 9.44) years. When characterizing school attainment, most caregivers had upper secondary education, while only 30% (n=10) had completed higher education. In addition, 30 (n=10) stated that they could not account for the hours spent being fully available to provide care. The remaining participants reported spending between 4 and 18 hours providing daily care. Most participants (n=17, 52%) reported that they were caregivers because there was no alternative.

**Table 1:** Sociodemographic data of caregivers who performed the preliminary evaluation of the massive open online course (n=33).

Variable		Participants, n (%)
**Gender**		
	Female	24 (73)
	Male	9 (27)
**Work status**		
	Full-time	11 (33)
	Part-time	4 (12)
	Does not work	18 (54)
**Relationship**		
	Child	19 (58)
	Mother or father	19 (58)
	Spouse	3 (9.1)
Cohabits with family member: yes		29 (88)
Support from family or friends: yes		24 (73)
**Dependence of the family member**		
	Chronic disease	7 (21)
	Aging process	10 (30)
	Mental disease	7 (21)
	Congenital deficiency	3 (9)
	Sequelae of accident or sudden event	6 (18)
Dependency condition: gradual evolution		21 (64)

### Material

The sociodemographic questionnaire allowed characterizing the participants according to age, marital status, education, cohabitation, work conditions, years of care, kinship, and care support.

The knowledge questionnaire with 20 questions was used to evaluate the contents of the course. The knowledge questionnaire was constructed for this purpose and pretested with caregivers. The questions focused on the signs, symptoms, and means of transmission of the SARS-CoV-2 infection and general measures of prevention, personal protection, and surveillance. They also focused on the frequency of change in positioning, benefits of transfer, hygiene care, prevention of pressure injuries and falls, measures promoting hydration and healthy eating, attitudes toward food refusal, and safe use of medicines and care with personal protective equipment. Each question had 4 possible answers, and each correct question was scored with 1 point. The questionnaire was administered before and 2 weeks after the completion of the MOOC.

The questionnaire to evaluate the acceptance of the MOOC was developed according to the technology acceptance model (TAM) [16]. It includes 14 questions about duration, the adequacy of the contents, language, sound, images, captions, whether the content was appropriate to the caregiver’s role, and the caregiver’s interest in applying what was learned. The responses were presented on a 5-point Likert ordinal scale of agreement, ranging from 1 (totally disagree) to 5 (totally agree). A section was also provided for comments and suggestions.

The Family Caregiving Factors Inventory (FCFI) [17] comprises 4 dimensions that evaluate caregiver resources, knowledge of care, caregiver expectations, and difficulties in caring tasks according to the health professional’s perspective. The knowledge of care dimension measures the degree of understanding that the caregiver has regarding the factors that can influence the patient’s health condition, environmental factors that can affect their safety, and factors that can interfere with their emotional and cognitive state; this dimension has 7 items and is evaluated on a 5-point Likert ordinal scale ranging from 1 (very poor) to 5 (very good). Difficulties in caring tasks may arise from the unpredictability of the condition of the recipient of care, lack of cooperation, uncontrollable external factors, dissimilarity between different caregivers, heavy physical work, or the permanent nature of care. This dimension includes 6 items evaluated on an ordinal scale varying from (1) not difficult to (5) very difficult. The caregiver resources dimension addresses the caregiver’s ability to achieve desirable care and may include self-skills and attitudes and the support of family and friends. This dimension has 7 items evaluated on an ordinal scale that varies between (1) usually not able to make correct judgments and (3) able to make correct judgments. The caregiver expectations dimension refers to whether the caregivers’ role is realistic. It includes 5 items, evaluated on a nominal scale that varies between realistic (1 point) and unrealistic (0 points). The results were standardized so that each dimension had a final score ranging from 0 to 100.

### Procedures

A sociodemographic questionnaire and a knowledge questionnaire were applied at the beginning of the study. After attending the MOOC, the caregivers were again asked to complete the knowledge questionnaire and the TAM. Data collection was performed by a nurse from the caregivers’ association mentioned above. The study was conducted from November to December 2020. The association nurse offered technical support and guidance to participants during this period through face-to-face meetings, telephone calls, or videoconferences. The objective was to clarify any doubts about the registration process and access to the platform.

### Data Analysis

Quantitative data were analyzed with SPSS (version 28; IBM Corp). Univariate analysis was performed through measures of central tendency and dispersion. In addition, a 2-tailed *t* test for paired samples was applied to compare the median scores obtained in the knowledge test before and after the MOOC. Finally, Pearson correlation was used to analyze the association between outcome variables.

### Ethics Approval

The study was approved by the Escola Superior de Enfermagem do Porto (ESEP) (ADHOC_1434/2020). The participants were informed of their right to withdraw from the study at any time. In addition, participants signed informed consent forms and were informed of the confidentiality of the data. This study followed the principles of the Declaration of Helsinki.

## Results

Before attending the MOOC, participants presented an average score of 14.94 (SD 2.72) and 16.52 (SD 2.28) after viewing the course; this difference was statistically significant (*t*_32_=4.180; *P*<.001) and shows increased knowledge about the topics addressed. The questions with the highest number of incorrect answers were related to prevention measures, such as personal protection attitudes when providing care, measures for packaging of care items and storing masks, safe mask use on public transportation, prevention of pressure injuries, and how to dispose of medicines. Although the caregivers considered the questions easy and accessible, they were still acquiring new knowledge. Through the analysis of the FCFI questionnaire, most caregivers showed adequate knowledge to respond to the care challenges and good personal and social resources to assist their family members. However, the respondents also pointed to some difficulties associated with the caregiver’s role. The caregivers’ expectations were rather dispersed, but overall, they were realistic and adapted to the situation.

The FCFI correlation matrix showed a moderate correlation between knowledge and care resources (*r*=0.56; n=30; *P*=.001) and a moderate but negative correlation between knowledge and difficulties (*r*=−0.54; n=30; *P*=.002). No statistical significance was found for the correlation between knowledge and expectations.

In addition, no statistical significance was found between the FCFI and the satisfaction scale or with specific knowledge from the MOOC. The assessment of satisfaction with the use of technology following the TAM model is shown in Table 2.

The caregivers’ assessments of the MOOC were mostly positive, suggesting that it was useful, clear, and had understandable information. This opinion was also shared by the caregivers, who said they usually had difficulties in understanding the information transmitted by health technicians. The course framework was considered appropriate, emphasizing the adequateness of the illustrative videos, the well-paced and clear voice, and the graphics. Also, the added value of this resource for support and decision-making on the care provided to the dependent was highlighted. Additionally, the benefits of this type of initiative were identified from the comments of the participants, who reported that the health services–targeted approach helped in their self-esteem and self-confidence.

After the validation of the MOOC, it was made available to the public throughout the year 2021 via the Nau platform. It was accessed by 2231 participants, who successfully completed 39,544 activities, which included viewing videos and filling out quizzes. The participants predominantly comprised individuals identifying as female (n=1716, 77%). The majority were aged 35 to 54 years. In terms of educational attainment, 52% (n=1116) had a higher education or secondary education qualification. Regarding the geographic distribution of the participants, the majority resided in Portugal. Nevertheless, there were participants from other countries, such as Switzerland, Brazil, Spain, France, Russia, and Venezuela.

**Table 2:** Assessment of satisfaction with the technology used.

	Score range	Median score (IQR)	Mean score (SD)
Course duration	2-5	4 (5-4)	4.35 (0.80)
Material provided	3-5	5 (5-4)	4.58 (0.56)
Course content	2-5	4 (5-4)	4.35 (0.66)
Relevance of themes	4-5	5 (5-4)	4.68 (0.48)
Language used	3-5	5 (5-4)	4.42 (0.67)
Video image	1-5	5 (5-4)	4.45 (0.93)
Video sound	1-5	5 (5-4)	4.39 (0.96)
Subtitle size	2-5	5 (5-4)	4.52 (0.72)
Response to expectations	3-5	4 (5-4)	4.35 (0.61)
Contribution to improving knowledge	3-5	5 (5-4)	4.55 (0.57)
Contribution to improving skills	1-5	5 (5-4)	4.42 (0.81)
Likelihood of retaking the course	1-5	4 (5-4)	4.39 (0.80)
Recommend the course	2-5	4 (5-4)	4.32 (0.65)
Overall assessment	1-5	5 (5-4)	4.45 (0.81)

## Discussion

### Principal Findings

This study sought to contribute to the empowerment of the caregiver by developing and validating a MOOC. Combining desk research with course recognition methodologies added more quality and effectiveness to the MOOC [18]. In addition, the opinion of people with a caregiver’s profile ensured that the proposed information, method, and evaluation tools are adapted to the target audience and are part of the procedure used in the evaluation of MOOCs [19-21].

MOOCs represent a product and a resource for quality training that have been introduced in the context of profound social transformation unleashed by the pandemic [22]. The European Commission emphasized the need to “rethink education,” with MOOCs being an important open and accessible training format [22]. However, a set of recommendations need to be considered in developing these MOOCs to ensure quality criteria are met [19,20,23]. For the design and development of this MOOC, all the quality-related recommended items were considered, such as course overview and introduction, learning objectives, assessment and measurement, instructional materials, learning activities and learner interaction, course technology, learner support, accessibility and usability, navigation, syllabus, instructor availability, and student input [24].

Therefore, it is crucial to analyze the impact of the course on improving the specific knowledge regarding the provision of care for the person dependent on self-care activities during the pandemic. The average score obtained before the course was 75% (n=25) correct answers, showing that knowledge before the course was already very high. After completing the course, the average score increased to 83% (n=27). Overall, the caregivers considered the course contents accessible and easy. The pedagogical evaluation of the knowledge acquired during the MOOC was described as an aspect that deserves attention [25]. As stated in other types of training, many variables influence the results. If participants consider that the questions are not difficult, it could lead to the perception of fewer gains. Conversely, if participants perceive the questions as difficult, it may discourage them. Notably, external and internal motivation is decisive for a better willingness to learn; however, these factors have not been evaluated. Although the results showed a significant improvement in knowledge, further detailed analysis of the questions with the highest percentage of incorrect answers is needed. In the diagnostic assessment of knowledge before the course, 6 questions registered over 25% (n=8) incorrect answers. After completing the course, 3 questions still showed a high percentage of wrong answers. The highest percentage of incorrect answers corresponded to the most difficult formulated questions. For example, one question addressed the care necessary to prevent pressure injuries with the following answer options: “(a) change position only when a reddish zone arises,” “(b) keep the bed linen well-stretched,” “(c) massage the reddish zones with alcohol,” and “(d) use an alternating pressure mattress to avoid changing the person’s position.” The correct validated answer for this answer was option b; however, after attending the course, many participants still chose option d. Advancements in differentiated technology prompted this answer, although the justification was unclear. During the course, the prevention of pressure injuries, such as by changing patient positioning every 2 hours, was identified as a major factor. Another question scoring the highest for incorrect answers was on the safe use of medicine, with the following answer options “(a) store the medicines in a dry and warm place,” “(b) store the medicines in the bathroom,” “(c) throwing medicines away in the trash past the expiration data,” and “(d) all the previous options are incorrect.” The correct validated answer for this was option (d). After completing the course, most participants chose the incorrect option c. However, returning these medicines to pharmacies was emphasized to ensure safe disposal. Another question was how to store face masks when they are removed, such as when eating, with the following answer options: “(a) store it in a paper bag or envelope, which must be disposed of after changing the mask,” “(b) store it in a tissue bag, which must be washed after changing the mask,” “(c) store it in a plastic bag with holes, which must be disposed of after changing the mask,” and “(d) all the above options are correct.” The correct answer was option d; however, most participants selected only the first option. This choice may have been influenced by some other sources because, at the time, option d was the most widely recommended in the media.

The results from the questionnaire to evaluate the acceptance of the MOOC following the TAM model showed that the caregivers were satisfied with the course, considering it useful, with clear and understandable information. Similar results were found in other studies [21,26,27]. Caregivers evaluated the course positively, and those with low literacy reported that the course was useful for caregivers since it delivered clear and understandable information. Implementing digital support tools requires the empowerment of informal caregivers in using them [28].

Participants described the adequateness of the course’s framework, the illustrative videos, the well-paced and clear voice, and the graphics. The added value of this resource to people’s lives was emphasized because it allowed them to make more informed decisions about the care to be delivered and the available resources. Participants also perceived that health professionals were attentive to and care about their needs. This aspect contributes to self-esteem and self-confidence for caregivers [28,29]. However, balancing the provision of care and the time needed to watch the videos was problematic for some caregivers. Therefore, nurses were key elements in ensuring project follow-up and continuity.

Despite these obstacles, 81% (n=27) of participants showed satisfaction with the web-based format of the course, especially during the pandemic. Similar results were found in other studies [21,30].

Less positive opinions were that the MOOC contents were more suitable for people with a physical disability than a cognitive impairment. Thus, guidelines that include customized options could make a strong contribution to improving the course [30].

The study’s positive results illustrate the caregivers’ commitment to their role, willingness to improve preparedness, and attentiveness to their social duties and responsibilities. The concept of e-learning, especially with web-based courses, is a new way to provide optimal learning opportunities, counseling, and support in health care [27]. In addition, the research team involved in the development of the MOOC is committed to the project and has raised awareness of the need to make adaptations in the teaching and learning process in an academic context and with caregivers. Similar results were obtained by other studies [7,8,27]. However, the acceptance and usability of digital technologies need to be further explored [31].

The results of the FCFI revealed that the caregivers’ current knowledge and resources to care for their dependent family members and their ability to perform the inherent tasks were adequate. This finding might be explained by the fact that this sample was recruited from a caregivers’ association, which aims to empower and support people in the caring process. These associations can be a valuable resource for the empowerment of the family caregiver because peer support helps people feel more capable of managing situations and seeking help and counseling [32,33]. The multidisciplinary team and the vital role of nurses are likely to contribute to excellent preparedness for the caregiver’s role. A recent study identified that nurses perform a set of essential activities with the caregiver in the identification of support needs, practical education, support in decision-making about treatment, and emotional support [33]. From our perspective, other factors that might have contributed to these results are related to the close relationship of the caregivers to the dependent people, which allowed greater proximity between the dyad of caregiver and care recipient. The progressive nature of the dependency also allowed for setting the pace of the adaptation to the new caregiver’s role, reducing difficulties in the caring process. Although more dispersed, the results also demonstrated the caregivers’ realistic expectations regarding the performance of their role. Again, sharing different experiences among the different caregivers and the support of health professionals can help caregivers attain mastery in care and meet physical, emotional, and comfort needs, among others. This sense of self-capability can also promote the caregiver’s resilience [33].

The use of virtual learning environments is becoming increasingly relevant, especially for individuals motivated to continuously learn outside more formal learning settings. Learning about specific content that can address real needs, such as health care situations, disease management, and their care, often serves as a motivating factor for using web-based tools, where people can learn at their own pace, frequently at no cost, and without the need for formal evaluation.

It seems reasonable that the use of MOOCs can improve health education. In fact, digital health literacy is already referred to as a superdeterminant of health [34]. Several studies have demonstrated the benefits of using MOOCs as promoters of learning about clinical aspects, serving as a tool that contributes to empowering individuals in making decisions about health issues [35,36].

Regarding the design of MOOCs, the authors suggest that their creators focus on building excellent content, ensuring good accessibility, as well as appropriate course assessment, covering correct information about their contents, in order to improve user satisfaction [37].

We believe that the findings in our study are aligned with these assumptions since the participants improved their knowledge and overall expressed satisfaction with the MOOC.

### Limitations of the Study

This study has some limitations. Due to constraints imposed by the pandemic, the convenience sample was recruited from a caregivers’ association. We believe that the caregivers who agreed to participate in the study were highly motivated to learn and develop skills, which may partially explain the positive outcomes, also evidenced before the course. Moreover, the fact that these caregivers belonged to an association likely contributed to their excellent preparedness for the role. Thus, further studies should focus on a random sample with community participants. Although external to the research team, problems and technical failures in the platform that supports the course were identified during the study, potentially affecting the overall assessment.

### Conclusions

The COVID-19 outbreak posed a tremendous challenge to health professionals and prompted them to find new strategies to provide optimal care. The changes that occurred, namely, the limitations in health services outreach and social isolation, precipitated the use of new technologies to respond to the educational needs of the population, particularly family caregivers. For example, the development of web-based MOOCs allowed empowering family caregivers to provide better care during the pandemic.

These positive results contribute to increased knowledge of family caregivers of home-dwelling–dependent persons. In addition, the MOOC received positive feedback from participants who integrated the project’s preliminary phase and was considered a valuable tool for informal caregivers with basic digital literacy.

This process enabled the course to be made available on the Nau platform. The initial approach showed that caregivers were poorly prepared for the performance of their role, specifically regarding protection measures during the pandemic, severely impacting the health and well-being of the most vulnerable people and creating an additional burden on the Portuguese National Health Service. Therefore, this MOOC constitutes an important tool to raise awareness and provide better training to informal caregivers. Moreover, this course can be used as an indirect resource to support health professionals and allied professionals working under unprecedentedly stressful conditions.

## Abbreviations

FCFI: Family Caregiving Factors Inventory
MOOC: massive open online course
TAM: technology acceptance model
WHO: World Health Organization

## Data Availability

The data sets generated and analyzed during this study are available from the corresponding author on reasonable request.
